# Functional analysis of the *theobroma cacao NPR1 *gene in *arabidopsis*

**DOI:** 10.1186/1471-2229-10-248

**Published:** 2010-11-15

**Authors:** Zi Shi, Siela N Maximova, Yi Liu, Joseph Verica, Mark J Guiltinan

**Affiliations:** 1 Huck Institute of Life Sciences, The Pennsylvania State University, University Park, PA 16802, USA; 2 The Department of Horticulture, The Pennsylvania State University, University Park, PA 16802, USA

## Abstract

**Background:**

The *Arabidopsis thaliana NPR1 *gene encodes a transcription coactivator (NPR1) that plays a major role in the mechanisms regulating plant defense response. After pathogen infection and in response to salicylic acid (SA) accumulation, NPR1 translocates from the cytoplasm into the nucleus where it interacts with other transcription factors resulting in increased expression of over 2000 plant defense genes contributing to a pathogen resistance response.

**Results:**

A putative *Theobroma cacao NPR1 *cDNA was isolated by RT-PCR using degenerate primers based on homologous sequences from *Brassica*, *Arabidopsis *and *Carica papaya*. The cDNA was used to isolate a genomic clone from *Theobroma cacao *containing a putative *TcNPR1 *gene. DNA sequencing revealed the presence of a 4.5 kb coding region containing three introns and encoding a polypeptide of 591 amino acids. The predicted TcNPR1 protein shares 55% identity and 78% similarity to *Arabidopsis *NPR1, and contains each of the highly conserved functional domains indicative of this class of transcription factors (BTB/POZ and ankyrin repeat protein-protein interaction domains and a nuclear localization sequence (NLS)). To functionally define the *TcNPR1 *gene, we transferred *TcNPR1 *into an *Arabidopsis npr1 *mutant that is highly susceptible to infection by the plant pathogen *Pseudomonas syringae *pv. tomato DC3000. Driven by the constitutive CaMV35S promoter, the cacao *TcNPR1 *gene partially complemented the *npr1 *mutation in transgenic *Arabidopsis *plants, resulting in 100 fold less bacterial growth in a leaf infection assay. Upon induction with SA, *TcNPR1 *was shown to translocate into the nucleus of leaf and root cells in a manner identical to *Arabidopsis *NPR1. Cacao NPR1 was also capable of participating in SA-JA signaling crosstalk, as evidenced by the suppression of JA responsive gene expression in *TcNPR1 *overexpressing transgenic plants.

**Conclusion:**

Our data indicate that the *TcNPR1 *is a functional ortholog of *Arabidopsis NPR1*, and is likely to play a major role in defense response in cacao. This fundamental knowledge can contribute to breeding of disease resistant cacao varieties through the application of molecular markers or the use of transgenic strategies.

## Background

Plants have evolved a complex network of defense responses, often associated with a response local to the site of infection [1-4]. In addition, defenses are also systemically induced in remote parts of the plant in a process known as systemic acquired resistance (SAR) [2,5,6]. Induction of the SAR pathway leads to heightened broad-spectrum resistance to secondary pathogen attacks by a variety of pathogens. Multiple studies in both monocots and dicots have shown that salicylic acid (SA) plays a central role as a signaling molecule in SAR [7-14]. Following pathogen attack, SA levels increase both locally and systemically in infected plants. In addition, SA is required for the induced expression of a set of pathogenesis-related (*PR*) genes [7,15-17].

NPR1 was originally identified by screening for mutants that were insensitive to SA (or its chemical analogs, 2,6-dichloroisonicotic acid (INA) or benzothiadiazole (BTH)) in *Arabidopsis *[7,18-20]. These screens identified a mutation designated as *Non-Expressor of PR1 *(*NPR1*). Studies that followed further documented that *npr1 *mutants displayed reduced expression of *PR *genes upon SA treatment and were more susceptible to pathogens [7,18,20,21]. Conversely, when *NPR1 *was overexpressed, the resulting transgenic plants displayed increased resistance to pathogens, and were able to induce increased levels of *PR *genes in a dose-dependent fashion [22].

*NPR1 *encodes a protein containing ankyrin repeats and a BTB/POZ domain, both of which mediate protein-protein interactions in animals [23]. NPR1 shares homology with IκBα transcription inhibitors, which regulate the innate immunity response [21,24]. Recent work has shed light onto the mechanisms of NPR1 function [5,6,10,17,25-27]. *NPR1 *is constitutively expressed, and NPR1 protein is present as inactive oligomers in the cytoplasm of the cell. Upon SAR induction, the redox state of the cell is altered, resulting in the reduction of NPR1 to its active monomeric form. Monomeric NPR1 moves into the nucleus where it can affect the induction of *PR *genes. Although NPR1 itself has no DNA binding domains, it participates in the regulation of defense gene transcription via interactions with TGA transcription factors [16,28-33]. In *Arabidopsis*, two conserved cysteine residues (C82 and C216) have been shown to be essential to the oligomerization and cytoplasmic localization of AtNPR1 [25]. Mutation of these residues results in constitutive monomerization and nuclear localization of NPR1.

It is believed that NPR1 also plays a role in the jasmonic acid (JA) signaling pathway and mediates the crosstalk between SA-JA defense pathways to fine-tune defense responses [27,30,34-36]. SA-mediated defenses are mainly effective against biotrophic pathogens, whereas JA-mediated defenses are predominantly efficient against necrotrophic pathogens and herbivorous insects. NPR1 mediates the antagonistic effect of SA on JA signaling by suppressing the expression of JA-responsive genes upon combined treatment of SA and methyl jasmonate (MeJA) [34].

A growing body of evidence has revealed that the salicylic acid dependent, NPR1-mediated defense pathway is also conserved in other plant species across wide phylogenetic distances. Two *NPR1-like *genes have been characterized from *Vitis vinifera *(grapevine) [14]. When translational fusions of the proteins encoded by the two genes with GFP were transiently expressed in *Nicotiana benthamiana *leaves, the proteins were localized predominantly to the nucleus and triggered the accumulation of pathogenesis-related proteins PR1 and PR2. In addition, the silencing of a tomato *NPR1-like *gene leads to increased bacterial growth upon *Ralstonia solanacearum *infection in tomato [12]. In tobacco, the suppression of *NPR1-like *gene leads to increased susceptibility to tobacco mosaic virus [8]. Similarly, overexpression of the apple *MpNPR1 *gene in transgenic apple plants resulted in the up-regulation of *PR *genes and enhanced resistance to bacterial and fungal pathogens [37]. In wheat, the expression of *Arabidopsis NPR1 *confers resistance to Fusarium head blight in susceptible cultivar Bobwhite [13]. Major efforts have been made to study the SA and NPR1-dependent pathway in rice, the model monocot plant. Treatment of rice plants with the salicylic acid analog probenazole results in enhanced resistance against rice blast fungus [38]. In addition, rice plants expressing bacterial salicylate hydrolase (*nahG*) are unable to accumulate salicylic acid and display increased susceptibility to rice blast [39]. Overexpression of the *Arabidopsis NPR1 *gene in rice leads to enhanced resistance to the bacterial pathogen *Xanthomonas oryzae *pv. *oryzae *[9]. An orthologue of *NPR1 *has been isolated from rice (*OsNPR1*/*NH1*), and the overexpression of *OsNPR1 *in rice leads to enhanced resistance to both bacterial and oomycete pathogens [40]. Moreover, *OsNPR1 *is able to complement the *Arabidopsis npr1-1 *mutant [11]. Like AtNPR1, OsNPR1 is also constitutively expressed and localizes to the cytoplasm. Treatment of rice cells with a reducing agent resulted in the movement of OsNPR1 into the nucleus. Similar to *Arabidopsis *NPR1, mutation of the corresponding cysteines (C82 and C216) in OsNPR1 also resulted in constitutive nuclear localization [11]. Thus, it appears that the mechanisms of SA-dependent, NPR1-mediated defense response likely evolved very early in the emergence of the plant kingdom.

*Theobroma cacao *L, (cacao) is a small tropical tree species endemic to the Amazon rainforest of South America. Cacao seeds are harvested and processed into cocoa beans and chocolate, providing an income for millions of small-holder farmers in West Africa, Central and South America, the Caribbean, Malaysia, Indonesia and other tropical areas. Pathogens are a major problem for cacao production, causing annual crop losses estimated at 30-40% [41]. In its center of diversity, the Amazon basin, cacao is susceptible to several potentially devastating pathogens, such as *Moniliophthora perniciosa*, the causal agent of witches' broom disease, *Moniliophthora roreri*, the causal agent of frosty pod rot [41-45] and several *Phytophthora *spp., the causal agent of black pod disease [46,47]. Outside this region, cacao is susceptible to a number of opportunistic pathogens [48-50].

Several defense-related genes in *Theobroma cacao *have been identified through gene expression analyses after hormone treatments [46,47,51]. An endo-1,4-β-glucanase is induced by the application of ethylene, and a type III peroxidase and a class VII chitinase are induced by methyl jasmonate treatment in mature cacao leaves. Those genes are responsible for induced resistance to pests in cacao, though the responses to hormone induction are different depending on developmental stages. In addition, transgenic overexpression of a class I chitinase gene in cacao enhances foliar resistance against the fungal pathogen, *Colletotrichum gloeosporioides *[52]. Moreover, ESTs sharing sequence homology to known *PR *genes have been isolated from cacao [53-55]. Several of these genes have been shown to be up-regulated by treatment of plants with benzothiadiazole (BTH), the salicylic acid analog [53]. All together, recent evidence suggests that cacao may utilize SAR pathway during the defense response; however, the extent of conservation of the pathway in cacao is presently unknown.

In this paper, we report the isolation and characterization of an *NPR1 *homologue from the tropical tree, *Theobroma cacao*. We show that *Theobroma cacao NPR1 *(*TcNPR1*) shares similar functions as *Arabidopsis NPR1*. It is able to partially complement the *Arabidopsis npr1-2 *mutation in transgenic *Arabidopsis *plants in a leaf infection assay and translocate into nucleus upon SA induction in the same manner as the endogenous *Arabidopsis *NPR1 protein.

## Results

### Isolation of a putative *TcNPR1 *gene

Degenerate PCR was utilized to clone the full length cDNA of *Theobroma cacao NPR1 *(*TcNPR1*). The degenerate primers were designed based on the alignment of NPR1 homologs from *Arabidopsis*, *Brassica *and *Carica papaya *and cDNA from cacao genotype Scavina6 (SCA6) leaf was used as template. A fragment of 1776 bp was isolated, cloned into pGEM sequencing vector and sequenced to reveal an intact coding sequence of the expected length and with high homology to the *Arabidopsis NPR1 *gene.

**Figure 1:**
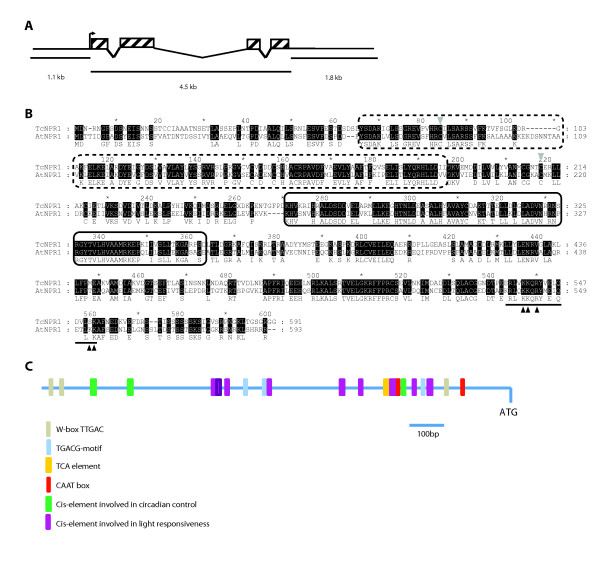
**Gene and protein structures of *Theobroma cacao NPR1***. **A**. Diagram of *TcNPR1 *gene structure. Boxes with diagonal stripes represent exons. Diagonal lines represent introns. The arrow represents the start site of transcription. The sizes of the promoter region, coding and the 3'-untranslated (UTR) regions of *TcNPR1 *are indicated. **B**. Alignment of AtNPR1 and TcNPR1 proteins. Protein alignment was carried out by ClustalW. Residues blocked in black are identical in both sequences. Numbers refer to the amino acid position in AtNPR1 protein. BTB/POZ and ankyrin repeats domains are highlighted by dashed line box and solid line box, respectively. Two of the conserved cysteines (C82 and C216 in AtNPR1) are shown with grey triangles. The potential nuclear localization signal identified in *Arabidopsis *is underlined. Amino acids demonstrated to be critical for AtNPR1 nuclear translocation are indicated with black triangles. **C**. Schematic representation of predicted cis-acting regulatory DNA element in cacao *TcNPR1 *promoter region. A 1.1 kb DNA fragment upstream of start codon was analyzed by querying the PLACE and PlantCare databases. The colored blocks represent different cis-elements as indicated.

A genomic fragment containing a putative *TcNPR1 *gene was obtained by screening Clemson University Genomics Institute (CUGI) cacao BAC library using the putative cacao *TcNPR1 *cDNA clone as probe. Two BAC clones were found to contain the *TcNPR1 *gene: 2K13 and 11K17. The genomic sequence of *TcNPR1 *was isolated by primer walking sequencing from known sequence using clone 2K13. A similar strategy was performed to sequence a region of 1.1 kb containing the promoter sequence upstream of ATG start codon. The full sequence consisted of a 4.5 kb genomic region of *TcNPR1 *containing 1.1 kb promoter, four exons and three introns (depicted in Figure 1A), which is similar to the genomic structure of *AtNPR1*.

### *Arabidopsis *and cacao NPR1 protein sequences are highly similar

Conceptual translation of the cacao NPR1 protein revealed that it consists of 591 amino acid residues, only two amino acids shorter than AtNPR1. Alignment of the AtNPR1 and TcNPR1 protein sequences revealed that they are highly similar to each other (55% identity, 74% similarity). Both the *Arabidopsis *and cacao *NPR1 *genes encode predicted proteins that share a number of structural features (Figure 1B). Each has a BTB/POZ domain near its N-terminal end (dashed line box) which shares 65% identity. Similarly, an ankyrin repeat region (solid line box) is present in both proteins which shares about 72% identity. In other ankyrin containing proteins, these domains have been shown to play roles in protein-protein interactions [16,23,56,57]. In the AtNPR1 protein, the BTB/POZ domain has been shown to function in homo-dimerization of NPR1, and the ankyrin repeat region mediates interactions with TGA transcription factors [58]. In addition, two cysteine residues (C82 and C216 in AtNPR1), which have been shown to play a role in the redox regulated activation and nuclear localization [25], are also conserved in TcNPR1 (Figure 1B. grey triangles). In fact, the AtNPR1 and TcNPR1 proteins share eleven conserved cysteine residues, suggesting that they share a similar structural conformation. The C-terminal region of AtNPR1 has been shown to contain a nuclear localization signal (NLS) that directs NPR1 monomers into the nucleus upon induction [59]. Five basic amino acids in this region function directly in this role (Figure 1B, black arrows). Four out of five of these basic amino acids are identical in TcNPR1, suggesting that TcNPR1 may also contain functional nuclear localization sequences. These similarities in protein structure suggest that *TcNPR1 *gene may also share the same function as *AtNPR1 *during plant defense response.

### Cacao NPR1 gene promoter contains putative SA regulatory elements

We analyzed the 1.1 kb promoter region of the TcNPR1 gene (Figure 1C) using plant cis-acting regulatory elements databases PLACE http://www.dna.affrc.go.jp/PLACE/[60] and PlantCare http://bioinformatics.psb.ugent.be/webtools/plantcare/html/[61,62]. Although a potential CAAT box was found 290 bp and 140 bp upstream of the ATG start codon, we did not observe an element resembling a TATA box. This is not surprising, as recent studies of core promoter regions in both plants and animals suggest that only 24%-29% of genes contain TATA-like elements [63,64]. A variety of other regulatory elements were also found. Several elements known to regulate inducibility by salicylic acid were found, such as the AS-1 element (TGACG). TGACG motifs were found involved in transcription activation by SA and this element was previously shown to be required for the SA-induced expression of *PR1 *[65]. In addition, there were multiple copies of the W-box (TTGAC), an element similar to the AS-1 element, which was also found in promoter of *AtNPR1*. W-box was shown to be the binding site for SA-induced WRKY DNA binding proteins [66], and was required for the SA induction of the tobacco (*Nicotiana tabacum*) class I chitinase gene [67]. All of the information suggests that the *TcNPR1 *gene might be regulated by SA in a manner similar to *AtNPR1*. Interestingly, several cis-elements involved in light responsiveness and circadian control are also presented in the *TcNPR1 *promoter, suggesting that *TcNPR1 *might be also regulated by light.

### Basal and induced expression of *TcNPR1 *in cacao tissues

**Figure 2:**
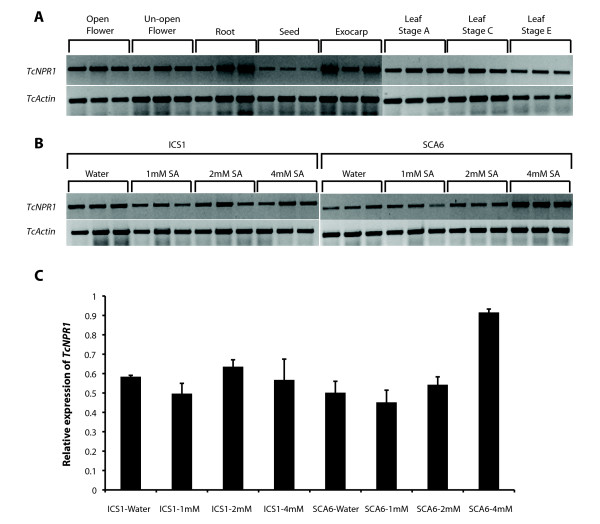
**Gene expression analysis of *TcNPR1 *in cacao**. **A**. Expression of *TcNPR1 *in various cacao tissues. Total RNA samples were collected from open flowers, unopened flowers, roots, seeds, exocarp and three different leaf developmental stages from youngest to oldest (A, C and E) from cacao genotype Scavina6 (SCA6). Semi- quantitative RT-PCR was performed and cacao *actin *(*TcActin*) was used as cDNA loading control. **B**. Expression of *TcNPR1 *in cacao leaf tissue after salicylic acid (SA) treatment. Semi-quantitative RT-PCR was performed with cDNA from stage C leaves of two different cacao genotypes ICS1 (left panel) and SCA6 (right panel), sampled 24 hrs after SA treatment in three different concentrations (1 mM, 2 mM and 4 mM). Water-treated samples served as a control and *TcActin *was used as cDNA normalization control. **C**. Calculated average relative gene expression levels from **B**. Gel images were quantified by ImageQuant and expression of *TcNPR1 *was normalized to *TcActin*. Expression levels are presented as the means ± standard errors of three biological replicates.

Semi-quantitative RT-PCR was performed to illustrate the basal expression level of *TcNPR1 *in various cacao tissues of Scavina6, including leaves from stage A (young/expanding), C (expanded/soft), E (mature/hardened), open flowers, unopened flowers, roots, seeds and fruit exocarps. *TcNPR1 *transcript was detected in all tissues tested (Figure 2A), an expression pattern similar to the *Arabidopsis *gene, however, the basal level of expression varies among different tissues. The expression of *TcNPR1 *was relatively high in the younger leaves (stage A and C) and lower in the later stages of development (stage E). The lowest expression of *TcNPR1 *in all tested tissue was observed in seeds whereas the expression was relatively high in fruit exocarps. In flowers, expression of *TcNPR1 *was higher in open flowers than in unopened ones. The expression of *TcNPR1 *in roots was at a moderate level, comparable to that in flowers and younger leaves.

### Induction by SA

Since it is well-characterized that *NPR1 *transcript accumulation can be increased by SA treatment of *Arabidopsis *leaves, we tested if *TcNPR1 *can respond to exogenous SA in the same manner. We applied various concentrations of SA to stage C leaves of two genotypes, Scavina6 and ICS1, which differ in their resistance to witches' broom disease (Scavina6 is more resistant) [68]. In *Arabidopsis*, the *NPR1 *gene is induced approximately 2-3 fold 24 hrs after treatment of leaves with 1 mM SA [2,69]. Semi-quantitative RT-PCR was employed to demonstrate the induced level of *TcNPR1 *24 hours after SA application (Figure 2B). To quantify the expression of *TcNPR1 *after SA treatment, we measured the fluorescence intensity of ethidium bromide stained DNA fragments irradiated with UV light using a high-sensitivity camera and ImageQuant software. Data were normalized to the expression level of an *actin *control. The results presented in Figure 2B showed that there was no significant change of *TcNPR1 *expression upon 1 mM, 2 mM and 4 mM SA treatment in ICS1 compared to water control. However, in the Scavina6, there was a statistically-significant 2-fold increase of *TcNPR1 *at 4 mM SA induction, though there was no change upon 1 mM and 2 mM SA treatment.

### Complementation of *Arabidopsis npr1-2 *mutant

To assess the function of TcNPR1, we placed the cacao *TcNPR1 *gene under the control of the E-12 omega promoter and introduced it into the *Arabidopsis npr1-2 *mutant to test if it can restore the mutant phenotype. One of the well characterized phenotypes of this mutant is the lack of SA-dependent activation of the *PR1 *gene [18,21]. The *PR1 *gene is thought to encode a protein active in defense response and has been used as a marker of SA pathway activation in many studies and in different plant species.

**Figure 3:**
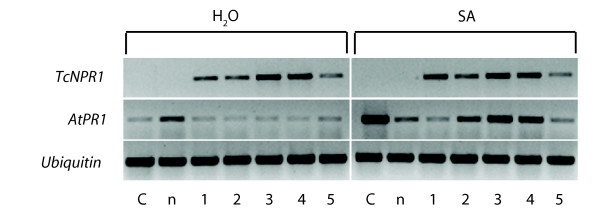
**Gene expression of *TcNPR1 *and *AtPR1 *in transgenic *Arabidopsis npr1-2 *lines**. Semi-quantitative RT-PCR was performed with cDNA prepared from the leaves of 4-week-old plants of wild type (C), *npr1-2 *(n) and 5 independent transgenic *npr1-2 *mutant lines overexpressing *TcNPR1 *(1-5). *TcNPR1 *and *AtPR1 *expression were evaluated 24 hrs after 1 mM SA treatment. Water-treated control leaves (left panel) from each genotype were also analyzed. *Arabidopsis Ubiquitin *(*AtUbiquitin*) expression was assayed as a non SA-induced, cDNA loading control.

Five independent *TcNPR1 *transgenic lines, wild type *Arabidopsis *Col-0 along with the *npr1-2 *mutant were sprayed with 1 mM SA, and the expression of *TcNPR1 *and *AtPR1 *was determined by semi-quantitative RT-PCR 24 hr after induction. Five transgenic lines all showed heterologous *TcNPR1 *expression with varied expression levels (Figure 3). As expected, there was no significant up-regulation of the transgene after SA treatment because *TcNPR1 *was expressed constitutively from the E12-Ω promoter. The *Arabidopsis PR1 *gene showed a very large induction after SA treatment in wild type *Arabidopsis *Col-0 (Figure 3), but there was no up regulation in the *npr1-2 *mutant, which is consistent with previous report [69]. There was a small increase in *PR1 *expression in the mutant treated with water, which could be expected from plant to plant biological variation. However, the *PR1 *gene expression level did not change after SA treatment, as expected for the *npr1-2 *mutant. We observed a moderate induction of the *PR1 *gene in 3 out of 5 transgenic lines (Line 2, 3 and 4), though the level of induction was not as high as in wild type Col-0. No *PR1 *gene induction was observed for transgenic lines 1 and 5. These results suggest that the *TcNPR1 *gene can at least partially complement the *Arabidopsis npr1*mutant and act to mediate SA dependent *PR1 *gene expression in *Arabidopsis *leaves but it may not act as efficiently as the endogenous NPR1 itself.

**Figure 4:**
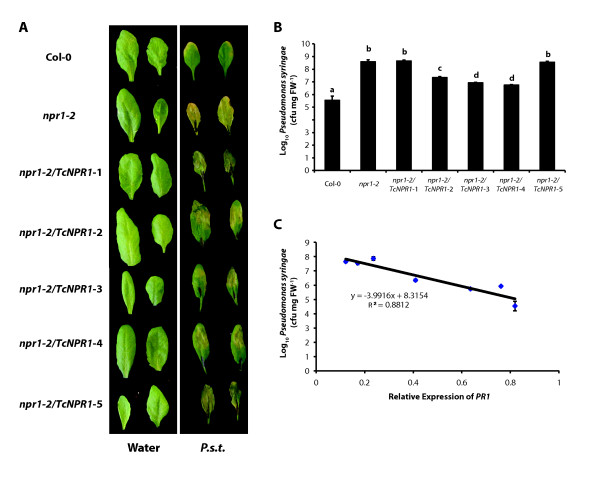
***Pseudomonas syringae *infection assay of transgenic *Arabidopsis npr1-2 *mutant lines**. **A**. Disease symptoms on leaves of Col-0, *npr1-2 *and five independent lines of *npr1-2 *plants transformed with *TcNPR1 *(*npr1-2*/*TcNPR1*) inoculated with *Pseudomonas syringae *pv. tomato DC3000 (*P.s.t*.) (OD_600 _= 0.002) at three days post inoculation and on leaves of the same seven genotypes infiltrated with water as a control treatment. **B**. Growth of *P.s.t*. in leaves from Col-0, *npr1-2 *and five individual transgenic lines (*npr1-2*/*TcNPR1*). Three days after inoculation, leaf disks were collected and bacterial titers were measured. Data represents the means ± standard errors of three biological replicates, each containing three leaf disks from three individual plants. Letters above the histogram indicate statistically significant differences among genotypes (P < 0.01) using the single factor ANOVA. **C**. Correlation of bacterial growth with relative *AtPR1 *expression level. Average growth of *Pseudomonas syringae *pv. tomato DC3000 (**Figure 4B**) and average *AtPR1 *gene expression (**Figure 3**) were evaluated in leaf tissue of Col-0, *npr1-2 *mutant and five transgenic lines expressing *TcNPR1*. Data was plotted and analyzed by liner regression analysis.

Another phenotype of the *Arabidopsis npr1 *mutation is increased pathogen growth after bacterial infection of leaves [18,21,69]. To test if *TcNPR1 *overexpression in *npr1-2 *mutant can complement the mutant disease susceptible phenotype, we infected leaves from 5 transgenic lines with *Pseudomonas syringae *pv. tomato DC3000 (*P.s.t*.) by syringe infiltration. The results indicated that the *npr1-2 *mutant was more susceptible than Col-0 (Figure 4A) three days after inoculation, exhibiting yellow necrosis similar to previous results [69]. Three transgenic lines overexpressing the *TcNPR1 *gene and exhibiting SA dependent *PR1 *up-regulation partially restored induced resistance compared to the control *npr1-2 *mutant (Figure 4A). Although several yellow necrotic spots were displayed on leaves of the transgenic plants, they did not exhibit severe necrosis or senescence. However, the other two transgenic lines, line 1 and 5, showed necrosis all over the leaves and the tissues were wilted. Water infiltration served as a control to demonstrate that the injection of water alone did not damage the tissues.

To quantify the disease symptom, bacterial assays were carried out to measure the titer of bacterial on infected leaves. The levels of bacterial in infected *npr1-2 *mutant leaf disks increased more than 250 fold as compared to Col-0 controls (Figure 4B). The three transgenic lines overexpressing the *TcNPR1 *gene (Line 2, 3 and 4), which exhibited significant up-regulation of the *PR1 *after SA treatment, showed a 30 to 100 fold reduction of bacterial growth compared to the *npr1-2 *mutant. There was no significant change in bacterial growth rates in leaf disks of the other two transgenic lines tested (Line 1 and 5). To assess the relationship between the level of SA-dependent induction of *PR1 *and the degree of bacterial growth in the transgenic lines, we plotted the values as depicted in Figure 4C. A significant negative correlation between SA dependent gene induction and bacterial growth was observed (R^2 ^= 0.88), suggesting that the resistance conferred by *TcNPR1 *is via the SA dependent resistance pathway and further supports our hypothesis that TcNPR1 plays a similar function to *Arabidopsis *NPR1 in plant defense response.

### Nuclear translocation of *TcNPR1 *after SA induction

**Figure 5:**
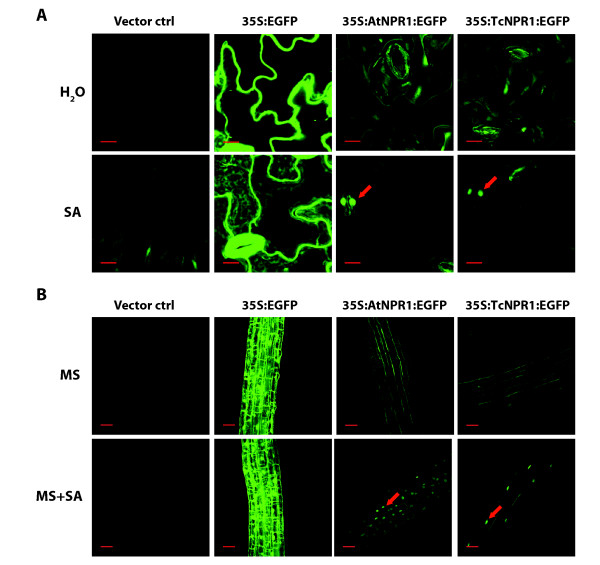
**Nuclear localization of TcNPR1-EGFP in transgenic *Arabidopsis *plants in response to SA**. **A**. Confocal images of EGFP fluorescence in *Arabidopsis *leaves of 4-week-old soil-grown plants 24 hrs after H_2_O (upper images) or 1 mM SA (lower images) treatment. All images were taken at the same magnification and exposure times. Arrows indicate the accumulation of green fluorescence in guard cell nuclei after SA treatment. Scale bar, 10 μm. **B**. Confocal images of EGFP fluorescence in *Arabidopsis *roots from 10-day-old seedlings grown on MS (upper images) or MS supplemented with 0.5 mM SA (lower images). All images were captured using the same exposure settings. Arrows indicate the accumulation of EGFP in nuclei of root cells after SA treatment. Scale bar, 30 μm. Samples from transgenic plants generated with pCAMBIA1300 (vector ctrl) was used as negative control and samples from transgenic plants expressing 35S:EGFP served as positive control in **A **and **B**.

Another hallmark of AtNPR1 function is its nuclear localization in response to treatment with SA [2,25,59,70,71]. To determine if TcNPR1 can also translocate into the nucleus in response to SA in a manner similar to *Arabidopsis *NPR1, we created transgenic *Arabidopsis *plants containing a TcNPR1-EGFP translational fusion and observed the subcellular localization of the fusion protein using confocal microscopy (Figure 5). This construct (35S:*TcNPR1:EGFP*) was stably transformed into the *npr1-2 *mutant and we observed the localization of EGFP fusion protein before and 24 hrs after SA treatment in both leaf and root cells of four independent transgenic lines. We observed no EGFP fluorescence in negative control plants transformed with the identical vector lacking the TcNPR1-EGFP fusion gene (Figure 5A and 5B). As an additional control, transgenic plants overexpressing EGFP without a fusion to TcNPR1 were imaged, and we observed strong fluorescence in both cytoplasm and nucleus with no localization changes after SA treatment. A final control consisted of a construct designed for the overexpression of the *Arabidopsis *NPR1 protein translationally fused to EGFP (35S:*AtNPR1*:*EGFP*). Consistent with the findings of others [25,59], we observed an extremely strong nuclear translocation of the fusion protein in leaf guard cells and in root cells 24 hrs after SA treatment.

The TcNPR1-EGFP fusion protein appeared to be evenly distributed in cytoplasm of leaf guard cells from water-treated 4-week-old soil grown plants, however, the protein accumulated moderately in guard cell nucleus 24 hours after SA application (Figure 5A, red arrow). Similarly, a modest level of nuclear translocation could also be observed in the root cells from 10-day-old seedlings grown on MS medium supplemented with 0.5 mM SA (Figure 5B). Although protein translocation of TcNPR1 is of lesser extent than observed with the *Arabidopsis *NPR1-EGFP protein based on reduced nuclear fluorescence observed in TcNPR1-EGFP transgenic plants, our results taken together indicate that TcNPR1, like *Arabidopsis *NPR1, can translocate into nucleus after SA induction and participate in the induction of defense related gene expression.

### *TcNPR1 *and SA-JA crosstalk

**Figure 6:**
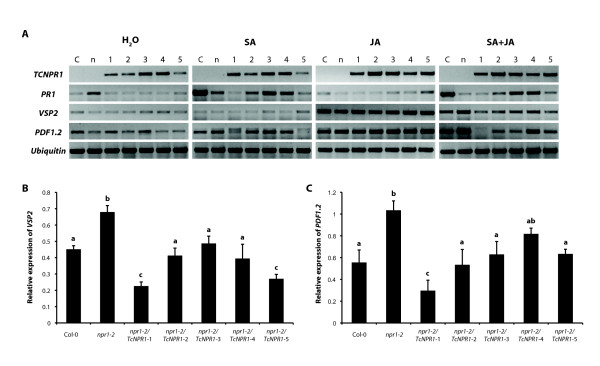
**Gene expression of SA- and JA-responsive genes in transgenic *Arabidopsis npr1-2 *mutants**. **A**. Semi-quantitative RT-PCR was performed with cDNA prepared from leaves of 4-week-old plants of wild type(C), *npr1-2 *(n) and 5 independent transgenic *npr1-2 *mutant lines overexpressing *TcNPR1 *(1-5). The expression of *TcNPR1*, *AtPR1*, *AtVSP2 *and *AtPDF1.2 *was evaluated 48 hrs after treatment with water control, 1 mM SA water solution alone, 0.1 mM MeJA alone in 0.015% Silwet L-77 and the combination of 1 mM SA and 0.1 mM MeJA in 0.015% Silwet L-77. *AtUbiquitin *was used as a cDNA loading and normalization control. **B**. The intensity of *AtVSP2 *and *AtPDF1.2 *RT-PCR gel bands in **Figure 6A **were quantified by ImageQuant software for total pixel intensity and the expression levels were normalized by *AtUbiquitin*. The bar charts represent the means ± standard errors of relative expression value of *AtVSP2 *and *AtPDF1.2 *following 48 hrs treatment of SA-MeJA combination of three biological replicates. Letters above the bar chart indicate statistically significant differences among genotypes (P < 0.05) determined by single factor ANOVA.

It has been previously demonstrated that *Arabidopsis *NPR1 can mediate the antagonism between SA and jasmonic acid (JA) by suppressing JA-responsive genes [27,34,35], suggesting that it plays an important role in fine tuning the cross-talk between different regulatory pathways. To explore the role of TcNPR1 in cross-talk, we tested the effect of SA and JA treatments on defense gene expression in wild type Col-0, *npr1-2 *mutant and five independent 35S:TcNPR1 transgenic *Arabidopsis *lines. Semi-quantitative RT-PCR showed that all five lines carrying the cacao transgene expressed *TcNPR1 *at moderate levels, and these did not change much during hormone treatments (Figure 6A). Exogenous application of 1 mM SA activated *PR1 *in Col-0 and three transgenic lines, but not in *npr1-2 *mutant. Additionally, 48 hrs after treatment with 0.1 mM methyl jasmonate (MeJA) in 0.015% Silwet L-77, two well established MeJA inducible genes (*VSP2 *and *PDF1.2*) were up-regulated in wild-type plant and in *npr1-2 *mutant, consistent with previous reports [34,72]. Two DNA bands were detected in some of the *PDF1.2 *PCR products, and we determined that the smaller molecular weight band resulted from cDNA amplification and the large fragment resulted from amplification of genomic DNA (data not shown). As predicted, all five 35S:TcNPR1 transgenic lines exhibited levels of increased *VSP2 *and *PDF1.2 *that were similar to those seen in Col-0 plants. Upon treatment with a combination of 1 mM SA and 0.1 mM MeJA in 0.015% Silwet L-77, *PR1 *was expressed at a level similar to seen when plants were treated with SA alone, indicating that MeJA had no effects on SA-responsive *PR1 *expression. Both *VSP2 *and *PDF1.2 *expressed at significantly lower levels in Col-0 compared to that in *npr1-2 *mutant after SA and MeJA combined treatment, demonstrating the function of AtNPR1 in antagonistic repression of JA-responsive genes. All five transgenic lines containing *TcNPR1 *gene displayed reduced expression levels of JA-responsive gene expression upon SA and JA combined treatment compared to *npr1-2 *mutant, suggesting that TcNPR1 can also mediate SA-JA cross-talk in a manner similar to AtNPR1.

To quantify the expression of *VSP2 *and *PDF1.2 *after the treatment of the combination of SA and MeJA, we measured the band intensity as above (Figure 6B). The data was normalized to an *Ubiquitin *control for loading effects. The relative expression levels of *VSP2 *and *PDF1.2 *were significantly decreased in *TcNPR1 *expressing transgenic lines compared to *npr1-2 *mutant (P < 0.05), a pattern similar to wild-type Col-0, suggesting that *TcNPR1 *restored the *npr1 *phenotype. These data support our hypothesis that TcNPR1 may play a role in mediating SA-JA cross talk as does *Arabidopsis *NPR1.

## Discussion

We have isolated an *NPR1 *homologous gene from the tropical tree, *Theobroma cacao*, and have generated transgenic *Arabidopsis npr1-2 *mutant lines overexpressing *TcNPR1*. All of our results are consistent with the hypothesis that *TcNPR1 *is a functional orthologue of the well characterized *Arabidopsis *gene. *TcNPR1 *complemented each of the major *Arabidopsis npr1-2 *mutant phenotypes that were tested. Over-expression of *TcNPR1 *in the *npr1-2 *mutant conferred *PR1 *up-regulation after SA treatment and increased resistance to *Pseudomonas syringae *pv. tomato DC3000 (Figure 3 and 4A, B). *TcNPR1 *was shown to be translocated into the nucleus in response to SA and to participate in SA-JA cross talk regulation (Figure 5 and 6). In our data, we found that transgenic lines 1 Line exhibited reduced complementation in SA-induced *PR1 *expression and disease resistance (Figure 3 and 4), while at the same time same two lines efficiently mediated crosstalk between SA and JA (Figure 6). In previous studies, the activation of defense related genes was shown to involve the nuclear translocation of NPR1 [59] while the crosstalk between SA and JA signaling was shown to be mediated by cytosolic NPR1 [34], thus it appears that very different mechanisms exist for these two functions of NPR1. The differential efficiencies of complementation of TcNPR1 we observed may reflect these different mechanisms. It is well known that positional effects (differential transgene transcription levels due to different genomic insertion sites in individual transgenic events) can have a large effect on protein expression levels. As suggested by RNA expression levels of the different TcNPR1 expressing transgenic lines (Figure 3), lines 1 and 5 may have lower protein expression than lines 2-4. It seems plausible that the differential complementation of the two NPR1 functions resulted from the differences in expression levels, potentially as a result of different protein accumulation levels in the cytosol vs nuclear compartments. Consistent with this idea, only the higher levels of expression seen in lines 2-4 was sufficient to complement the nuclear gene induction function, but the levels of expression were high enough in all lines to complement the cytosolic SA/JA crosstalk function.

In all, our results demonstrate a high degree of evolutionary and functional conservation of NPR1 from the Brassicales to the Malvales. NPR1 is also conserved in species as diverse as grapevine [14], tomato [12], apple [37], banana [73], cotton [74], tobacco [8] and rice [11]. This high degree of functional conservation suggests that NPR1 function evolved very early in the development of higher plants and that it plays a very critical role in plant development and reproductive success.

Little is known about the mechanisms of defense signaling in cacao. Our data suggests that the central mechanisms operative in *Arabidopsis *are likely to be conserved in cacao. At a minimum, our data suggests that the mechanisms and molecules that interact with NPR1 during SA and JA signaling and nuclear translocation are also conserved in cacao. If this were not the case, we would not expect the cacao NPR1 protein to function normally in *Arabidopsis*. However, the cacao protein in some cases only partially restored function of the *npr1 *mutant, which is likely the result of transgene expression level differences compared to the endogenous gene and/or partial molecular incompatibility with its interacting protein partners. It is possible that the binding affinities between the cacao NPR1-interacting proteins are reduced as compared to the endogenous *Arabidopsis *coevolved partners. Partial complementation has commonly been observed in heterologous complementation analysis in many other systems [75-77].

Further investigation is needed to explore the entire defense response pathway in *Theobroma cacao *and to understand the similarities and differences with *Arabidopsis *overall. For example, our expression data shows that *TcNPR1 *can be up-regulated only at 4 mM SA treatment but not at lower concentrations, which is higher than the optimal level of 1 mM in *Arabidopsis *as previously indicated [69]. It would be interesting to test the endogenous SA level of cacao and to determine dose response dynamics in various tissues and during different stages of development. Another area of interest is to identify and characterize the downstream targets of *TcNPR1 *and to compare them to the approximately 2,248 genes that are regulated by NPR1 during systemic acquired resistance in *Arabidopsis *[78]. Surveying these genes in cacao could reveal interesting differences in the defense responses unique to this tropical tree relative to *Arabidopsis*. Furthermore, *Arabidopsis *NPR1 has been shown to interact with several different proteins such as the TGA transcription factors [16,28,33,70,79]. Thus another area of interest is to isolate *TcNPR1 *interacting cacao proteins, which will further enhance our knowledge of this pathway in cacao. We are also interested in studying other NPR1-like genes of cacao and the recent completion of a draft cacao genome sequence has led to the identification of three additional NPR1-like cacao genes [80].

Plant diseases, especially pathogenic fungi, are estimated to cause about 30-40% yield loss on cacao annually [41,81], and thus disease resistance is of substantial interest to cacao breeders. Our findings can be utilized in several approaches to help develop varieties of cacao with enhanced disease resistance. The sequence of the *TcNPR1 *gene could possibly be used to develop molecular markers and probes that can be employed to select disease resistant varieties with specific allelic variations. Interestingly, the major quantitative trait locus (QTLs) for witches' broom disease resistance is tightly linked to the *TcNPR1 *gene [80], thus the *TcNPR1 *gene serves as a key candidate gene for generation of molecular markers that can be used for marker assisted selection of new disease resistant varieties. In addition, *TcNPR1 *expression levels could be modified in transgenic cacao varieties to develop broad-spectrum disease resistance. This approach has already been successful in several species but to our knowledge, has not yet been deployed in commercial production for any species. However, consumer and industry reluctance to accept transgenic plant technology remains a formidable barrier to development of any transgenic cacao varieties for commercialization.

## Conclusion

The isolation of the *TcNPR1 *gene and its heterologous complementation in *Arabidopsis *allowed us to rapidly characterize the function of this defense-related gene. The up-regulation of *PR1 *and increased bacterial resistance in transgenic *Arabidopsis npr1-2 *mutants strongly supported that *TcNPR1 *is a functional ortholog of Arabidopsis *NPR1*, and vital component in SA-dependent signaling pathway in *Theobroma cacao*. Our results provide potential opportunities to enhance disease resistance in this crop species through conventional breeding or biotechnological approaches. Further investigation is needed to identify the TcNPR1 interacting transcription factors and their downstream targets in cacao and to reveal further details of the molecular mechanisms of the role TcNPR1 plays as a central mediator of the plant defense response.

## Methods

### Full-length cDNA Cloning by Degenerate PCR

*NPR1 *cDNA sequences from *Arabidopsis *(U76707), *Brassica napus *(AF527176), and *Carica papaya *(AY550242) were aligned using the ClustalW program v1.8 [82]. Degenerate primers (TcNPR1dg-5', TATTGTCAARTCTRATGTAGAT; TcNPR1dg-3', GAARAAYCGTTTCCCKAGTTCCAC) were designed to regions highly conserved among all three sequences.

Total RNA was isolated from cacao leaves from variety Scavina6 as previously described [53]. Cacao leaf cDNA was synthesized using the SMART RACE cDNA Amplification Kit (Clontech Laboratories Inc., Mountain view, CA http://www.clontech.com/) according to the manufacturer's instructions.

PCR reactions were performed using cacao leaf 2.5 μl cDNA from first strand synthesis from SMART RACE cDNA Amplification Kit, 10 μl Redi-prime PCR mix (GeneChoice, Inc., Frederick, MD) and 5 μM of the above degenerate primers. Following denaturation (94° for 5 min.), PCR was performed for 32 cycles using the following condition (94° for 30 sec., 45° for 30 sec, 72° for 1 min.), followed by a 5 min. extension at 72°. PCR products were resolved on 1% agarose gels, purified with the GENECLEAN II Kit (Q-Biogene Inc., Solon OH) and cloned into the pGEM-T-Easy vector (Promega Corporation, Madison WI) according to the manufacturer's instructions. DNA sequencing was performed at the Penn State Genomics Core Facility using an ABI Hitachi 3730XL DNA Analyzer. The resulting clone was designated as pGEM-TcNPR1.

### Genomic DNA cloning by BAC library screening

*Theobroma cacao *BAC filter arrays constructed using genomic DNA from genotype LCT-EEN 37 were purchased from the Clemson University Genomic Institute http://www.genome.clemson.edu/. Filter arrays were blocked for 4 hours at 60°C in a solution containing 1% BSA, 1 mM EDTA, 7% SDS, and 0.25 M sodium phosphate. PCR generated *TcNPR1 *cDNA fragment labeled with ^32^P dCTP using the MegaPrimer DNA Labeling System (GE Healthcare, Buckinghamshire, UK) according to the manufacturer's instructions was added and hybridized overnight at 60°C. The next day, the filter arrays were washed twice in 2× sodium chloride/sodium citrate (SSC), 0.5% sodium dodecyl sulfate (SDS) for 20 minutes at 60°C. Radiographic imaging was performed via storage phosphor imaging (Molecular Dynamics, http://www.mdyn.com/). After filter alignment and clone number identification, a BAC clone (2K13) containing a putative *TcNPR1 *fragment was obtained from a frozen stock. The sequence of *TcNPR1 *genomic fragment was acquired by series sequencing of the BAC clone from ATG start codon. Sequencing primer was designed based on the *TcNPR1 *cDNA at the first round and following series primers were designed based on the known sequence resulting from previous sequencing. Introns were identified by aligning the genomic sequence and full length cDNA using SPIDEY software http://www.ncbi.nlm.nih.gov/spidey/. The same strategy was applied to clone the 1.1 kb promoter region upstream of the ATG. Forward and reverse sequencing was also performed to validate the sequence.

For sequence verification the *Arabidopsis *NPR1 protein sequence (At1g64280) and putative cacao NPR1 protein sequences (genbank accession HM117159) were aligned using the ClustalW program v1.83 [82]. The TcNPR1 protein sequence was analyzed for potential functional sites by querying the Simple Modular Architecture Research Tool (SMART) database http://smart.embl-heidelberg.de/.

### Semi-quantitative RT-PCR analysis of *TcNPR1 *expression in cacao tissues

Total RNA was isolated from Scavina6 leaves stages A, C and E (corresponding to stages YR, IG, MG respectively, as described in [83]), open flowers, unopened flowers, roots, exocarps and seeds as previously described [53]. For each tissue, three biological replicates were collected and analyzed. Cacao cDNA was synthesized in a final volume of 25 μl from 2 μg of total cacao RNA using M-MLV reverse transcriptase (New England Biolabs, Inc., Ipswich, MA). RNA and 0.5 μg oligo(dT) were added to sterile water to final volume of 18 μl. The mixture was then incubated at 70°C for 5 min, chilled on ice, which was followed by adding 10× reverse transcription buffer (New England Biolabs, Inc., Ipswich, MA), 0.1 M fresh made DTT and 10 mM dNTP. The mixture was further incubated at 42°C for 2 min, followed by incubation at 42°C for 1 hr with 10 units of reverse transcriptase MMLV (New England Biolabs, Inc., Ipswich, MA). The reaction was terminated at 70°C for 15 min.

Semi-quantitative RT-PCR was performed using intron-spanning primers for *TcNPR1 *(TcNPR1RT-5': ATGGATTCCCGTCTGGAACTTGGT; TcNPR1RT-3': TCTGGAGTGTCATTTCCTCCGCAT) and *TcActin *(CL33contig2 in Esttik Database http://esttik.cirad.fr/ used as an internal normalization and cDNA loading control (TcActinRT-5': AGCTGAGAGATTCCGTTGTCCAGA and TcActinRT-3': CCCACATCAACCAGACTTTGAGTTC). RT-PCR reactions were set up using 1 μl of 1/2 diluted cDNA and 5 μM of the *TcNPR1 *or *TcActin *primers. Titration of cycles was carried out and it was determined that the PCR amplification of *TcNPR1 *was within its linear range at 27 cycles using the following condition: 94°C for 30 sec., 56°C for 30 sec, 72°C for 1 min. Similarly, PCR of *TcActin *was performed under non-saturation conditions within the linear range (22 cycles at 94°C for 30 sec., 55°C for 30 sec, 72°C for 1 min). *TcActin *served as a cDNA loading control.

### SA treatment of cacao seedlings

The leaves of two to three-month old cacao plants generated by rooted cuttings from two different genotypes (ICS1 and Scavina6) were sprayed with salicylic acid (SA) dissolved in water at three different concentrations, 1 mM, 2 mM and 4 mM. Control plants were treated with water. Plants were grown in a greenhouse under conditions previously described [53] and leaf tissue from fully expanded young leaves (developmental stage C, corresponding to stage IG in [46]) was harvested at 24 hrs after treatment and frozen in liquid nitrogen. Total RNA was isolated and cDNA was synthesized as described above. For each genotype and each treatment, three biological replicates were collected. Semi-quantitative RT-PCR and expression analysis were performed to assay the levels of *TcNPR1 *expression as described above. The PCR products were analyzed on 1% agarose gel, stained with ethidium bromide. The expression values of *TcNPR1 *and *TcActin *were quantified using ImageQuant software (Molecular Dynamics, Amersham Bioscience) as described in [84] and relative expression of *TcNPR1 *was calculated by comparing with the expression of *TcActin*.

### Transgenic *Arabidopsis *mutant complementation assay

All binary plant transformation vectors were constructed by incorporating the genes of interest into pCAMBIA-1300 binary transformation vector containing plant selectable marker for hygromycin resistance [85].

Binary Vector p35S:TcNPR1 - The *TcNPR1 *coding sequence fragment was generated by PCR using pGEM-TcNPR1 as described above and included *Xma*I and *Not*I restriction sites at the 5'- and 3'-ends respectively (TcNPR1-5'-*Xma*I, CCCGGGATGGATAACAGAAATGGCTT; TcNPR1-3'-*Not*I, GCGGCCGCTTGCATTAGGCCTATGGTCTA). The fragment was cloned into pGEM T-Easy (Promega Corporation, Madison WI) according to the manufacturer's instructions and sequenced for integrity. The *TcNPR1 *coding sequence was then cloned into the *Xma*I and *Not*I sites of an intermediate cloning vector (pE2113) between E12-Ω promoter [86] and 35SCaMV terminator. A 3 kb restriction fragment containing *TcNPR1 *gene cassette was excised from pE2113 using *Pvu*II and ligated into the *Sma*I site of pCAMBIA-1300.

Ligations were performed overnight at 16° with 3Units of T4 DNA ligase (Promega Corporation, Madison WI).

Binary vector p35S:AtNPR1 - The *AtNPR1 *coding sequence fragment was generated by PCR using the *AtNPR1 *cDNA clone U13446 from Arabidopsis Biological Resource Center http://www.biosci.ohio-state.edu/~plantbio/Facilities/abrc/abrchome.htm and included *Nco*I sites at the 5'- and 3'-ends (AtNPR1-5'-*Nco*I, CCATGGACACCACCATTGATGGATTC; AtNPR1-3'-*Nco*I, CCATGGTCCGACGACGATGAGAGAGTTTACG). The PCR fragment was cloned into pGEM T-Easy and sequenced. The resulting intermediate plasmid was designated pGEM-AtNPR1. The *AtNPR1 *coding sequence was then excised by *Nco*I from pGEM-AtNPR1, and blunt-end cloned into pE2113 between E12-Ω promoter [86] and 35SCaMV terminator as *Xma*I and *Not*I fragment. Contently 3.1 kb fragment containing the *AtNPR1 *gene cassette was obtained by digestion with *Pvu*II, and blunt-end ligated into the *Sma*I site of pCAMBIA-1300.

Binary Vector p35S:TcNPR1:EGFP - The cassette of E12-Ω promoter and EGFP on the intermediate cloning vector pE2113 was cloned into *EcoR*I and *Hind*III sites of pCambia1300. The resulting binary vector was designated pXCGH. PCR generated *TcNPR1*fragment, including *Sma*I and *KpnI *sites at the 5'- and 3'-ends (TcNPR1-5'-*Sma*I, CCCGGGATGGATAACAGAAATGGCTT; TcNPR1_3'-*Kpn*I, GGTACCGACCGCCCCTACCACTACCAGTTAG) was first cloned into pGEM T-Easy (pGEM-TcNPR1-EGFP). The sequence was verified, the DNA fragment was excised with *SmaI *and *KpnI *and blunt ends ligated into the blunt-ended *NcoI *site of pXCGH positioned between the E12-Ω promoter and at the 5'end of the EGFP coding sequence to generate the binary vector p35S:TcNPR1:EGFP.

Binary vector p35S:AtNPR1:EGFP - The pGEM-AtNPR1 containing *AtNPR1 *coding sequence was digested with *Nco*I and the fragment was ligated into the *Nco*I site of pE2113 as described above. The 3.6 kb fragment containing the *AtNPR1*- EGFP fusion gene cassette was digested with *Sal*I and *Eco*RI and cloned into the *Sal*I and *Eco*RI sites of pCAMBIA-1300.

### *Arabidopsis *Transformation

The binary vectors described above were introduced into *Agrobacterium tumefaciens *strain AGL1 by electroporation, as previously described in [87]. *Arabidopsis *Col-0 plants were grown in a Conviron growth chamber (Model No. MTPS144) maintained at 22°C, under a 16:8::L:D cycle. Light intensity was maintained at 200 μM/m^2^·s with Octron 4100K Ecologic bulbs (Sylvania, Danvers MA). To increase the number of inflorescences, plants were cut back after bolting, and allowed to re-grow. The floral dip method was used to transform *Arabidopsis *as described previously [88]. Briefly, Agrobacterium cultures were grown at 25° on a platform shaker (200 rpm) to an OD_600 _= 1.2. Cells were centrifuged at 1,500 × *g *for 6 minutes and re-suspended in 300 mls of a solution containing 2.15 g L^-1 ^MS salts, 5% sucrose, 0.02% Silwet-77. The flowers were dipped in the solution for three seconds, domed to remain humidity and covered with black cloth. The cloth was removed the next day and plants were regularly watered until seed maturation.

Following seed set, seeds were collected from nine plants for each independent transgenic event. Seeds from 5 individual lines were soaked in 0.1% Tween-20 for 2 minutes and sterilized with 50% bleach for 10 minutes at room temperature. Seeds were then washed five times with 1 ml of sterile water. To select for transformants, seeds were planted on 1/2 MS media, agar plates (pH 5.7) supplemented with 25 μg ml^-1 ^hygromycin B. Plates were place in a Conviron growth chamber under the same light and temperature conditions as above. After 10 days, germinated seedlings were examined for leaf development and root elongation. Those seedlings that showed root elongation were transferred to soil and allowed to grow. Transformations were performed with the following vectors: p35S:TcNPR1, p35S:AtNPR1, p35S:TcNPR1:EGFP, and p35S:AtNPR1:EGFP constructed as described above, and control vectors p35S: EGFP (pGH00.0126, [89])and pCambia 1300.

### Salicylic acid (SA) *Arabidopsis *induction assay

Four week-old wild type *Arabidopsi*s Col-0 and *npr1-2 *mutants and five independent transgenic lines growing in soil were sprayed with 1 mM SA, along with water-treated control plants. Three biological replicates, each containing leaves from 5 individual plants were collected 24 hrs after treatment. Total RNA was isolated from treated and control samples using RNeasy plant mini kit (QIAGEN, Valencia CA). cDNA was generated as described above. Semi-quantitative RT-PCR was performed as described above to measure the expression of *TcNPR1 *and *AtPR1*. *Arabidopsis Ubiquitin *served as cDNA loading and normalization control. The following primer sets and conditions were employed:

TcNPR1-5': ATGGATTCCCGTCTGGAACTTGGT; TcNPR1-3': TCTGGAGTGTCATTTCCTCCGCAT (27 cycles of 94°C for 30 sec., 56°C for 30 sec., 72°C for 1 min). AtPR1-5': CTCGAAAGCTCAAGATAGCCCACA; AtPR1-3': CTTCTCGTTCACATAATTCCCACG (25 cycles of 94°C for 30 sec., 54°C for 30 sec., 72°C for 1 min). Ubiquitin-5': ACCGGCAAGACCATCACTCT; Ubiquitin-3': AGGCCTCAACTGGTTGCTGT (22 cycles of 94°C for 30 sec., 54°C for 30 sec., 72°C for 1 min). The conditions of PCR were determined by cycle titration to avoid saturating conditions. The relative expression levels were determined as described above.

### *Pseudomonas syringae *infection assay of *Arabidopsis *transgenic plants overexpressing *NPR1 *genes

*Pseudomonas syringae *pv. tomato DC3000 (*P.s.t*.) was grown on Difco Pseudomonas agar (PA) (Becton, Dickinson and Company, http://www.bdbioscience.com/) supplemented with rifampicin (100 μg ml-1) and kanamycin (25 μg ml-1) at 25°C for 48 hrs. Cells were scraped from plates using a bacterial inoculating loop and re-suspended in water. Plant infection assays and bacterial growth assays were carried out as described previously in [90]. Five individual transgenic *npr1-2 *mutant overexpressing *TcNPR1 *coding sequence were infected with *P.s.t*. at OD_600 _= 0.002. Briefly, three days after inoculation leaf disks from treated leaves of 2 independent replicate plants were pooled for a single sample. Data represents means ± SE (cfu/mg FW) of three biological replicates per treatment and statistical differences were determined by Single factor ANOVA analysis.

### Nuclear translocation of TcNPR1 in transgenic *Arabidopsis *plants

For observations of green leaves, four week-old soil-grown transgenic plants containing one of transgenes 35S:AtNPR1:EGFP, 35S:TcNPR1:EGFP, 35S:EGFP and plants transformed with empty binary vector pCambia 1300 were sprayed with either a 1 mM solution of SA in water or water. For root observations, control and transgenic seed were germinated on MS agar or MS agar supplemented with 0.5 mM SA [59] and seedlings were grown for 10 days. Leaves and roots were placed in a drop of water on a standard microscope glass slide and overlaid with a cover slip. The samples were imaged with an inverted Olympus FV1000 Laser Scanning Confocal Microscope (Olympus America Inc., Melville, NY). For imaging EGFP, tissues were excited with a blue argon laser (488 nm) and emission wavelengths of 500-600 nm were detected through a variable bandpass filter positioned in front of the photomultiplier tube. Tissues were observed using 40× and 10× objectives for leaf cells and root cells, respectively, each with numerical apertures and 1.15. FV10-ASW version 1.6 software (OLYMPUS, Pittsburgh, PA) was used to collect images, select slices, and create intensity projections over the Z axis.

### SA and JA combination treatment of *Arabidopsis *transgenic plants overexpressing *TcNPR*1

Four weeks old soil-grown wild type *Arabidopsi*s Col-0, *npr1-2 *mutants and five independent transgenic lines containing p35S:TcNPR1 were sprayed with a combination of 1 mM SA and 0.1 mM MeJA in 0.015% Silwet L-77. Plants treated with 1 mM SA alone in water, 0.1 mM MeJA alone in 0.015% Silwet L-77 and water with 0.015% Silwet L-77 served as control treatment. Three biological replicates each consisting of leaves from 5 individual plants were collected at 48 hrs after treatment, total RNA was isolated, cDNA was synthesized and semi-quantitative RT-PCR was performed as described above to determine the transcripts level of *TcNPR1 *and *AtPR1*. For expression analysis of *VSP2 *and *PDF1.2*, following primer sets and conditions were used to maintain the reaction in its linear amplification range. *VSP2 *Forward: TACGGTCTCGGCATCCGTTC; *VSP2 *Reverse: CCTCAAGTTCGAACCATTAGGCT (21 cycles of 94°C for 30 sec., 58°C for 30 sec., 72°C for 1 min). *PDF1.2 *Forward: TCATCATGGCTAAGTTTGCTTCCATC; *PDF1.2 *Reverse: TGTCATAAAGTTACTCATAGAGTGAC (27 cycles of 94°C for 30 sec., 60°C for 30 sec., 72°C for 1 min). The PCR products were analyzed on 1% agarose gel, stained with ethidium bromide. The expression values of *AtVSP2 *and *AtPDF1.2 *were quantified using ImageQuant software (Molecular Dynamics, Amersham Bioscience) as described in [84] and relative expression of two genes was calculated by comparing with the expression of *AtUbiquitin*.

### Accession numbers

Sequence data from this article can be found in the Arabidopsis Genome Initiative, GenBank/EMBL databases or Esttik database http://esttik.cirad.fr/ under the following accession numbers: At1g64280 (*NPR1*), At2g14610 (*PR1*), At5g24770 (*VSP2*), At5g44420 (*PDF1.2*), At3g52590 (*ubiquitin*), HM117159 (*TcNPR1*) and CL33contig2 (*TcActin*).

## Abbreviations

NPR1: non expressor of PR genes 1; SA: salicylic acid; INA: 2,6-dichloroisonicotic acid; BTH: benzothiadiazole; BTB/POZ: broad complex, tramtrack and bric a brac/pox virus and zinc finger; JA: jasmonic acid; PR: pathogenesis related; SAR: systemic acquired resistance; NLS: nuclear localization signal; MEJA: methyl jasmonate; VSP2: vegetative storage protein 2; PDF1.2: plant defensin 1.2; QTL: quantitative trait locus.

## Authors' contributions

ZS performed most of the experiments, *ie*, sequence analysis, gene expression studies, phenotypic analysis of transgenic *Arabidopsis *plants, confocal microscopy observations and drafted the manuscript. SNM participated in the design of the study, directed the transformation vector construction and transgenic lines generation, and participated in drafting of the manuscript. YL participated in transgenic *Arabidopsis *plants analysis and helped to analyze the sequence. JV cloned the *TcNPR1 *gene. MJG conceived the study, drafted the manuscript and gave advice on experimental design, data analysis and execution. All authors read and approved the final manuscript.
